# Supplementary material for: Energy Coupling and Stoichiometry of Zn2+/H+ Antiport by the Cation Diffusion Facilitator YiiP
Source: bioRxiv. 2023 Feb 23:2023.02.23.529644. Preprint. [Version 1] doi: 10.1101/2023.02.23.529644 (PMC9980050; doi:10.1101/2023.02.23.529644)
Supplement: Supplementary file 1 [file NIHPP2023.02.23.529644v1-supplement-1.pdf]

bioRxiv preprint doi: https://doi.org/10.1101/2023.02.23.529644; this version posted February 23, 2023. The copyright holder for this preprint (which was not certified by peer review) is the author/funder, who has granted bioRxiv a license to display the preprint in perpetuity. It is made available under aCC-BY 4.0 International license.

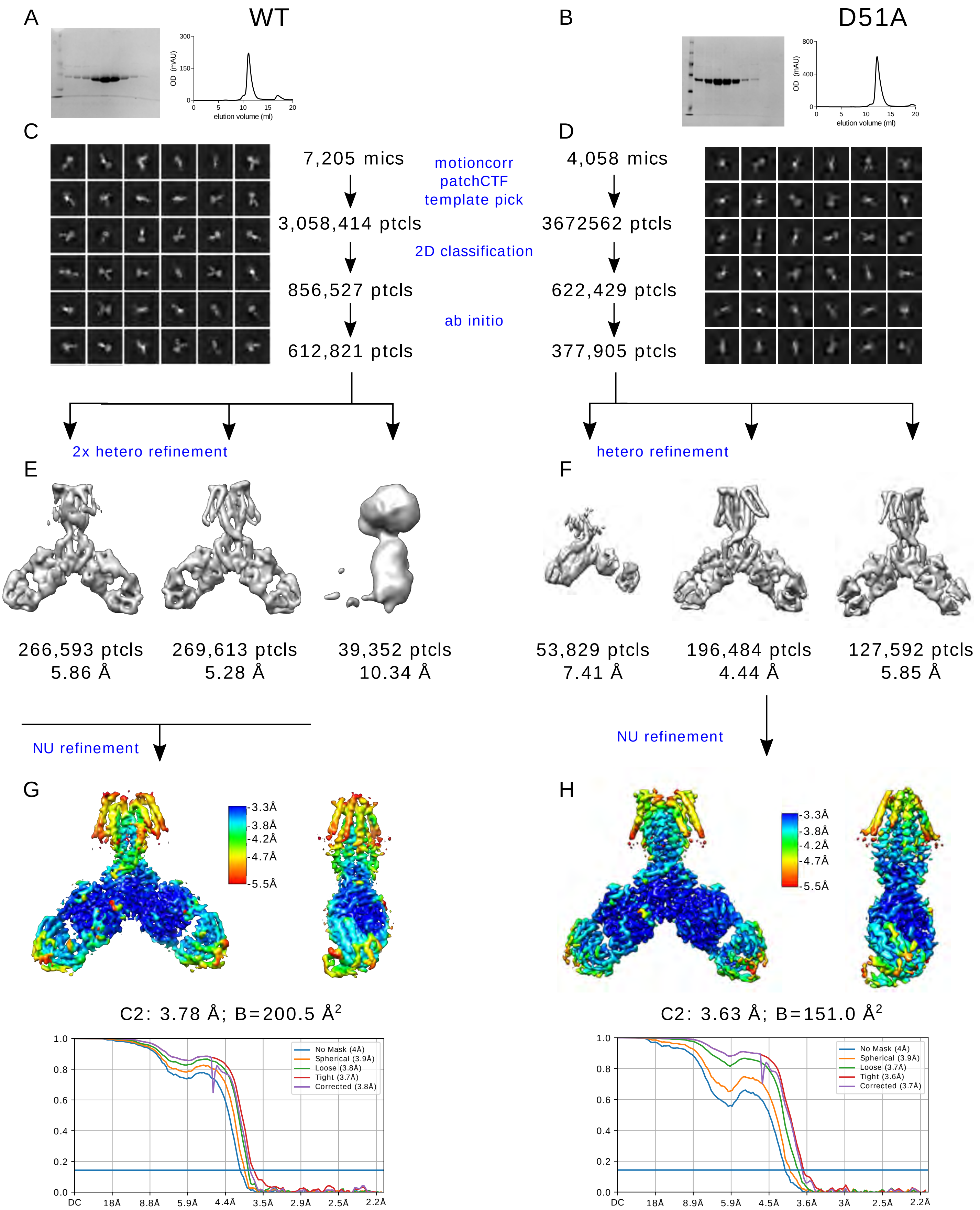

Suppl. Fig. 1

bioRxiv preprint doi: https://doi.org/10.1101/2023.02.23.529644; this version posted February 23, 2023. The copyright holder for this preprint (which was not certified by peer review) is the author/funder, who has granted bioRxiv a license to display the preprint in perpetuity. It is made available under aCC-BY 4.0 International license.

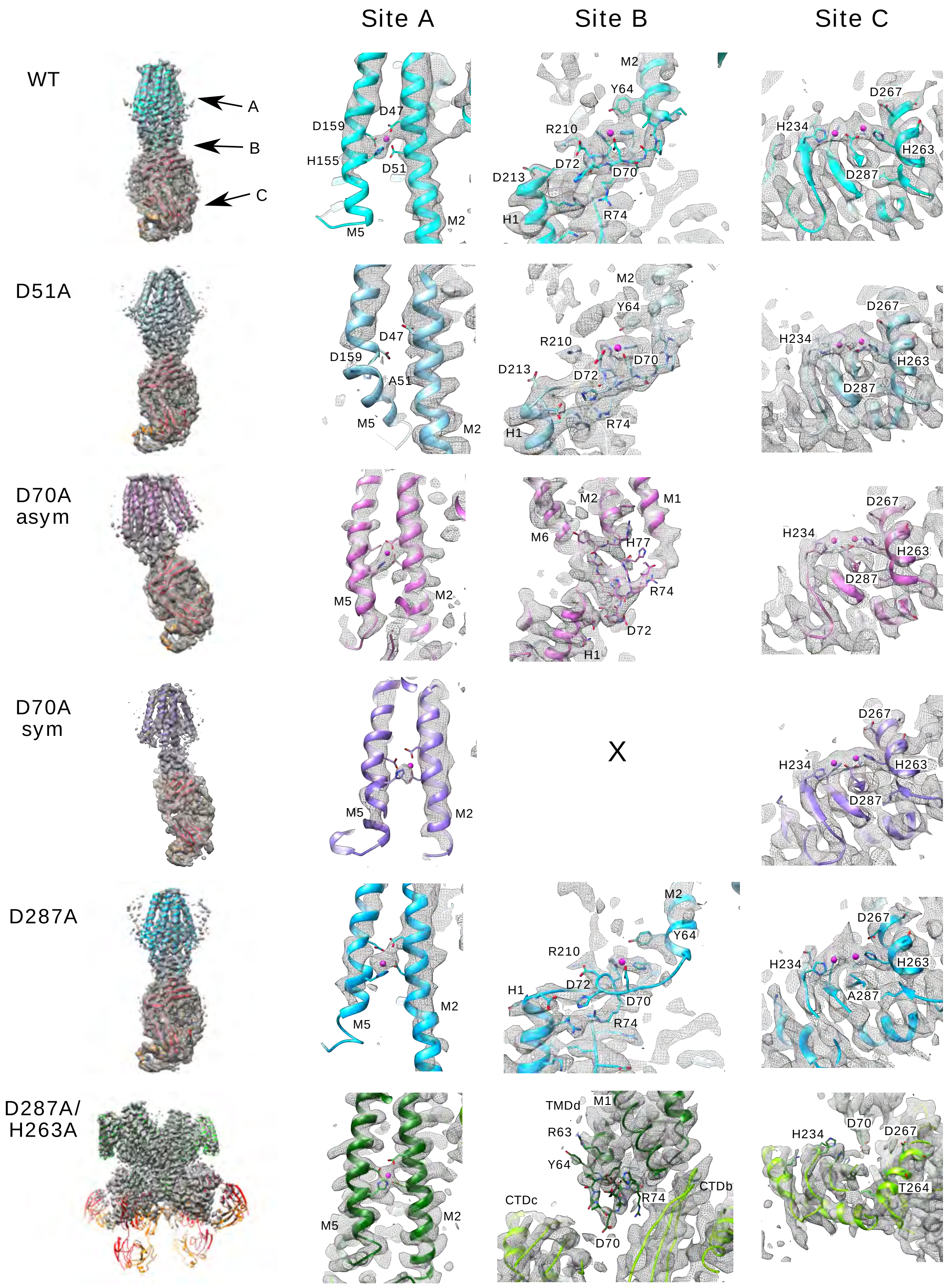

Suppl. Fig. 2

bioRxiv preprint doi: https://doi.org/10.1101/2023.02.23.529644; this version posted February 23, 2023. The copyright holder for this preprint (which was not certified by peer review) is the author/funder, who has granted bioRxiv a license to display the preprint in perpetuity. It is made available under aCC-BY 4.0 International license.

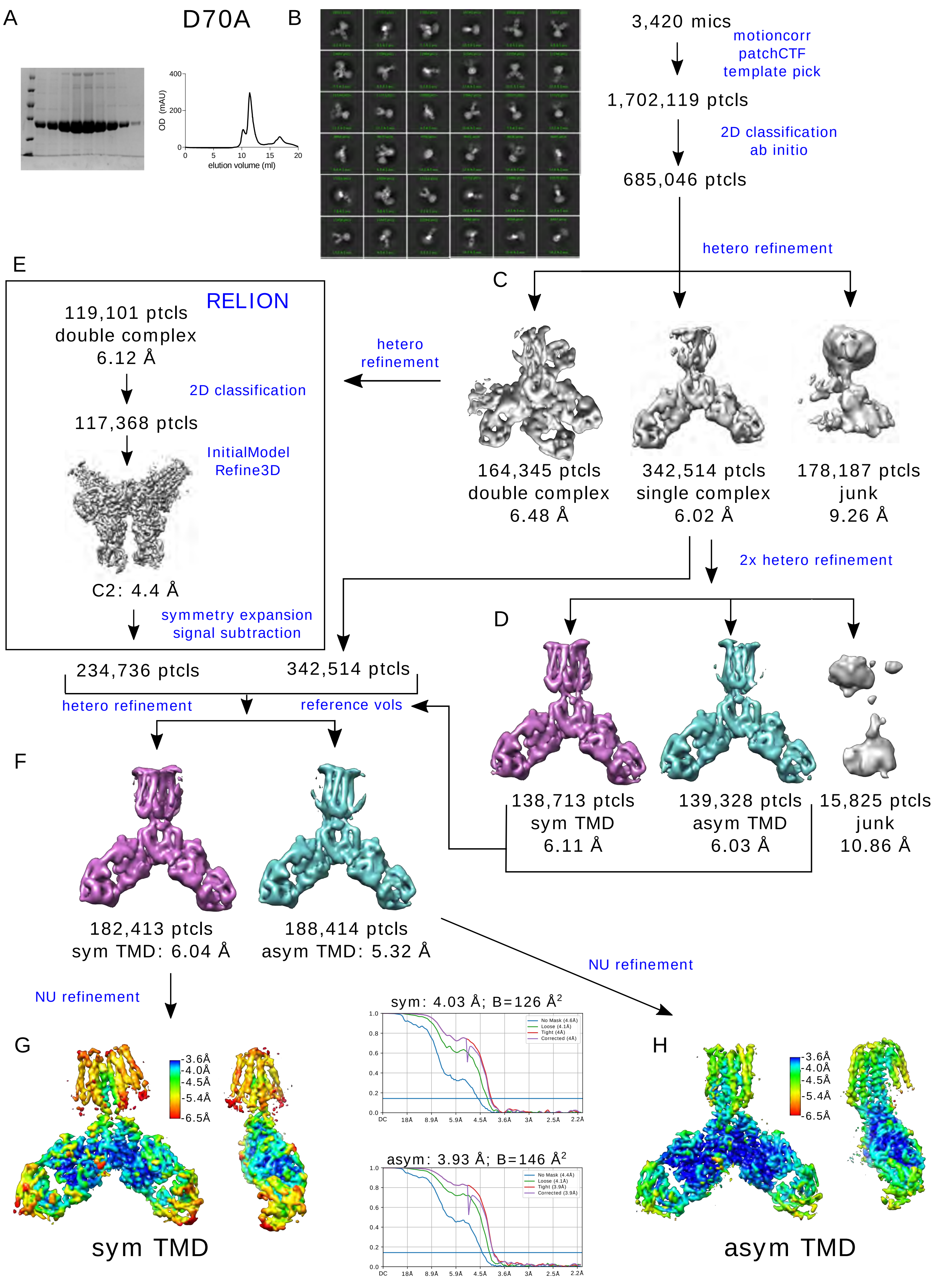

Suppl. Fig. 3

bioRxiv preprint doi: https://doi.org/10.1101/2023.02.23.529644; this version posted February 23, 2023. The copyright holder for this preprint (which was not certified by peer review) is the author/funder, who has granted bioRxiv a license to display the preprint in perpetuity. It is made available under aCC-BY 4.0 International license.

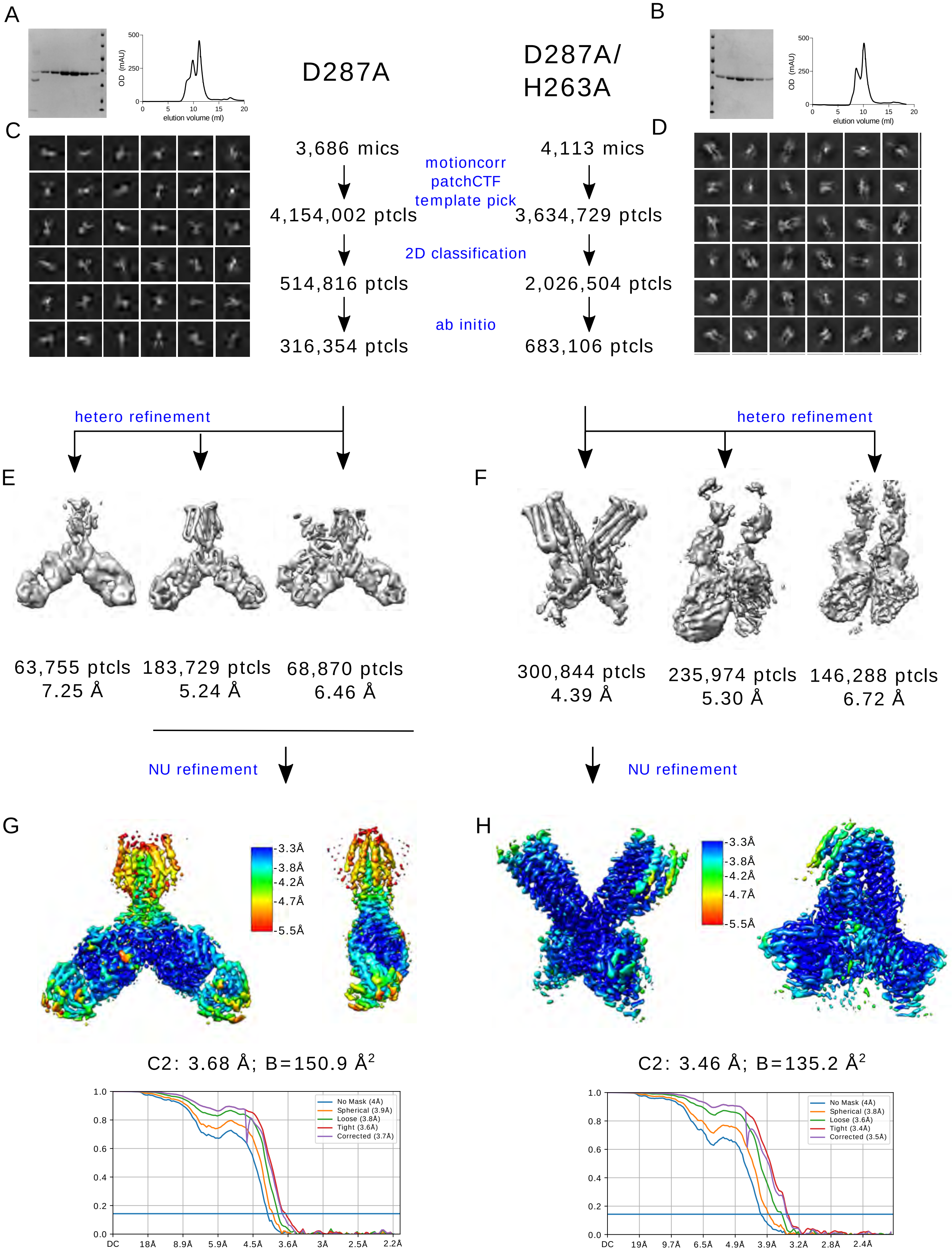

Suppl. Fig. 4

bioRxiv preprint doi: https://doi.org/10.1101/2023.02.23.529644; this version posted February 23, 2023. The copyright holder for this preprint (which was not certified by peer review) is the author/funder, who has granted bioRxiv a license to display the preprint in perpetuity. It is made available under aCC-BY 4.0 International license.

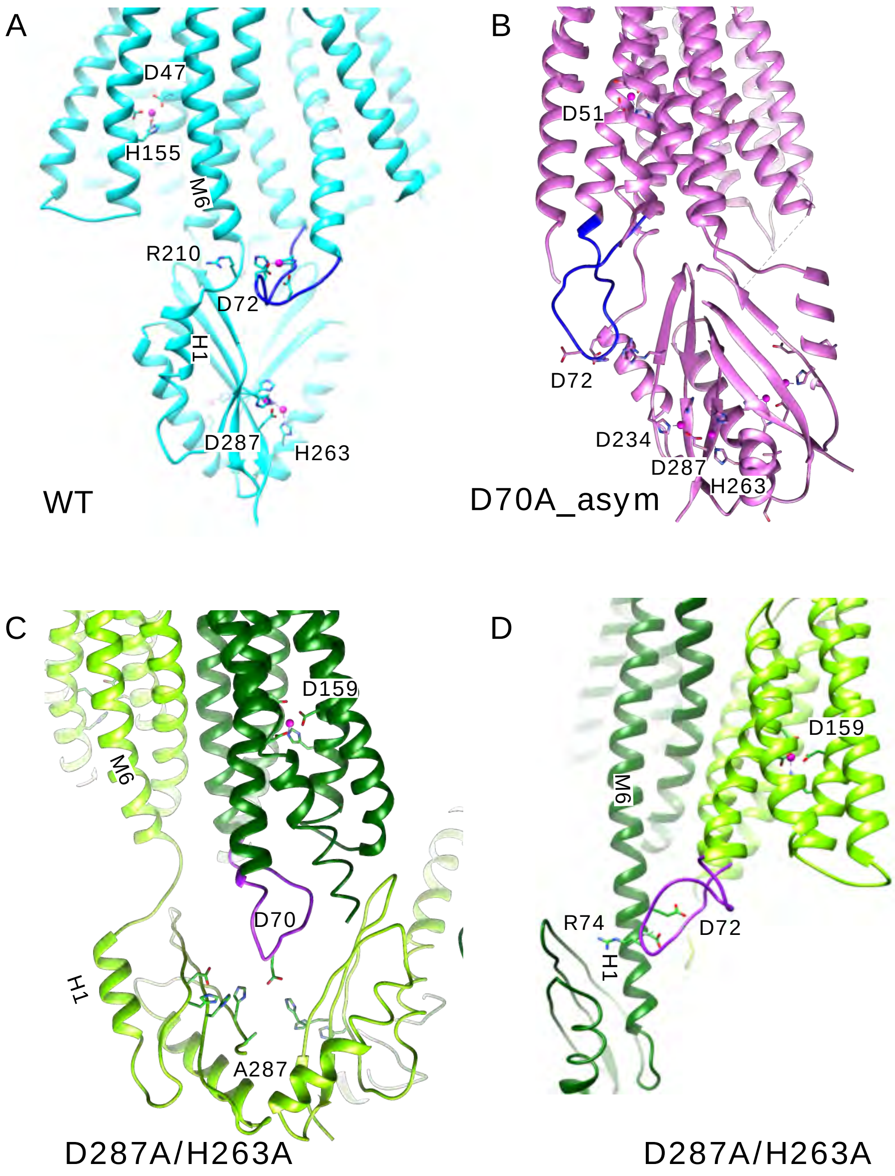

Suppl. Fig. 5

bioRxiv preprint doi: https://doi.org/10.1101/2023.02.23.529644; this version posted February 23, 2023. The copyright holder for this preprint (which was not certified by peer review) is the author/funder, who has granted bioRxiv a license to display the preprint in perpetuity. It is made available under aCC-BY 4.0 International license.

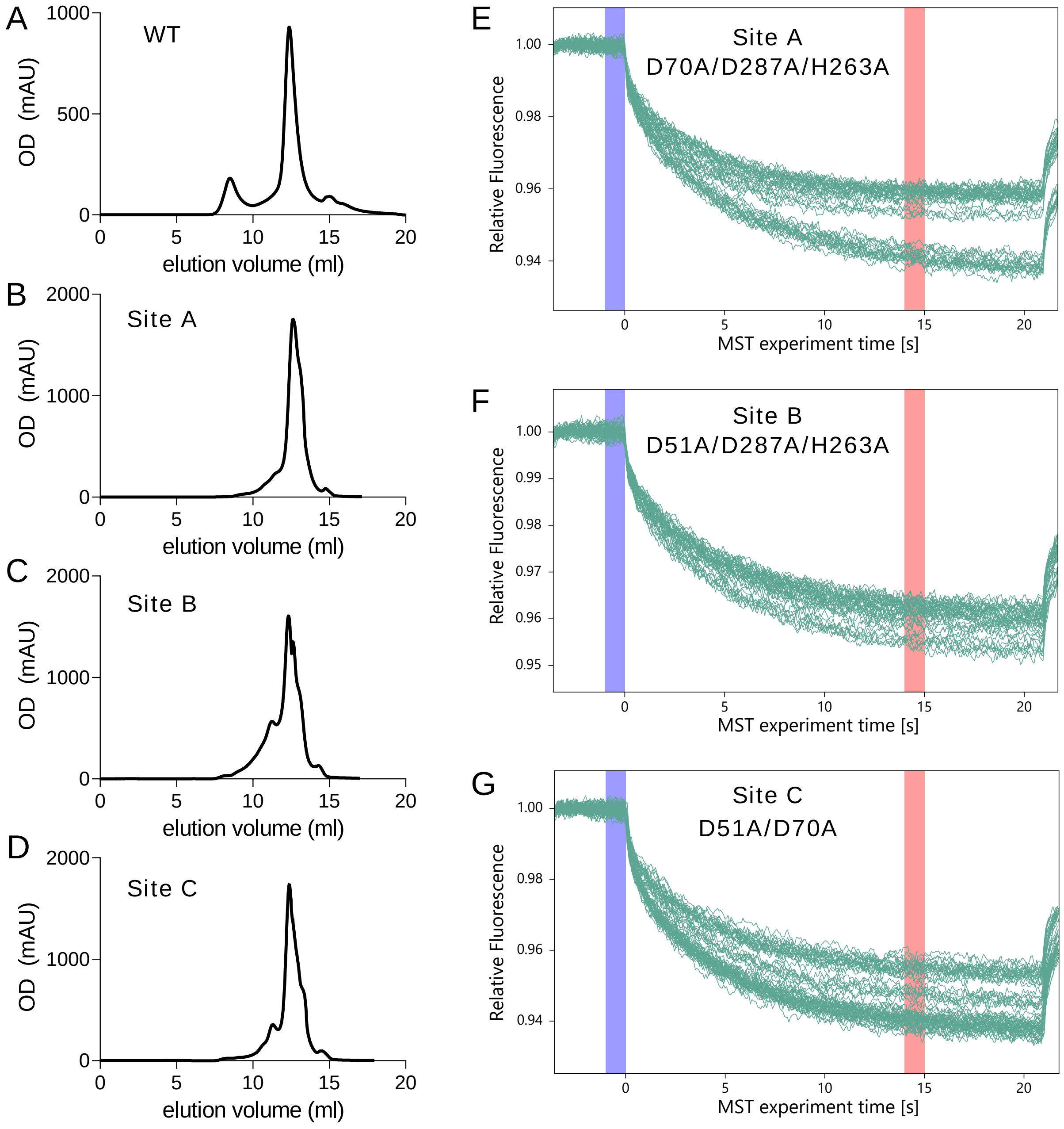

Suppl. Fig. 6

bioRxiv preprint doi: https://doi.org/10.1101/2023.02.23.529644; this version posted February 23, 2023. The copyright holder for this preprint (which was not certified by peer review) is the author/funder, who has granted bioRxiv a license to display the preprint in perpetuity. It is made available under aCC-BY 4.0 International license.

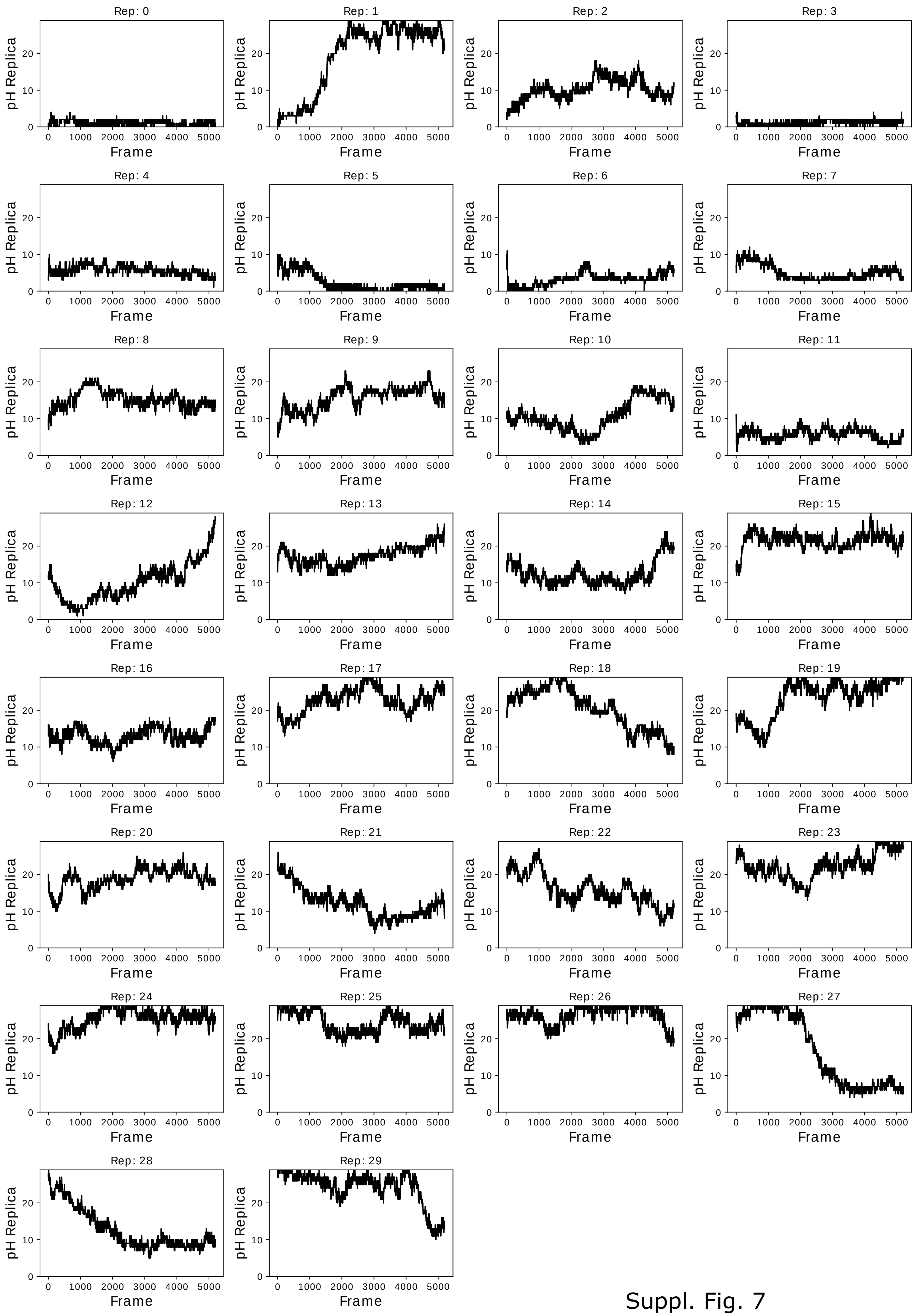

Suppl. Fig. 7

bioRxiv preprint doi: https://doi.org/10.1101/2023.02.23.529644; this version posted February 23, 2023. The copyright holder for this preprint (which was not certified by peer review) is the author/funder, who has granted bioRxiv a license to display the preprint in perpetuity. It is made available under aCC-BY 4.0 International license.

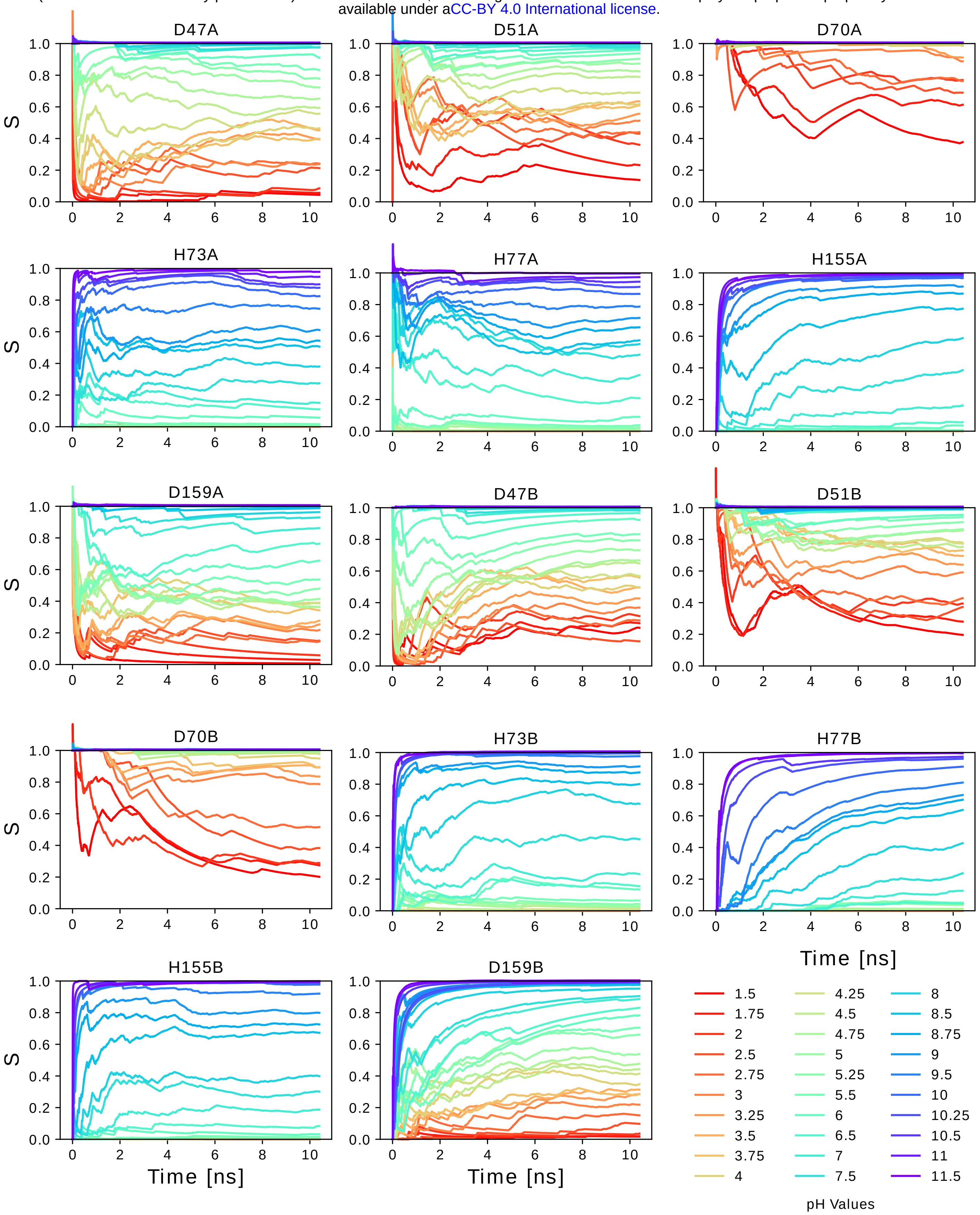

Suppl. Fig. 8

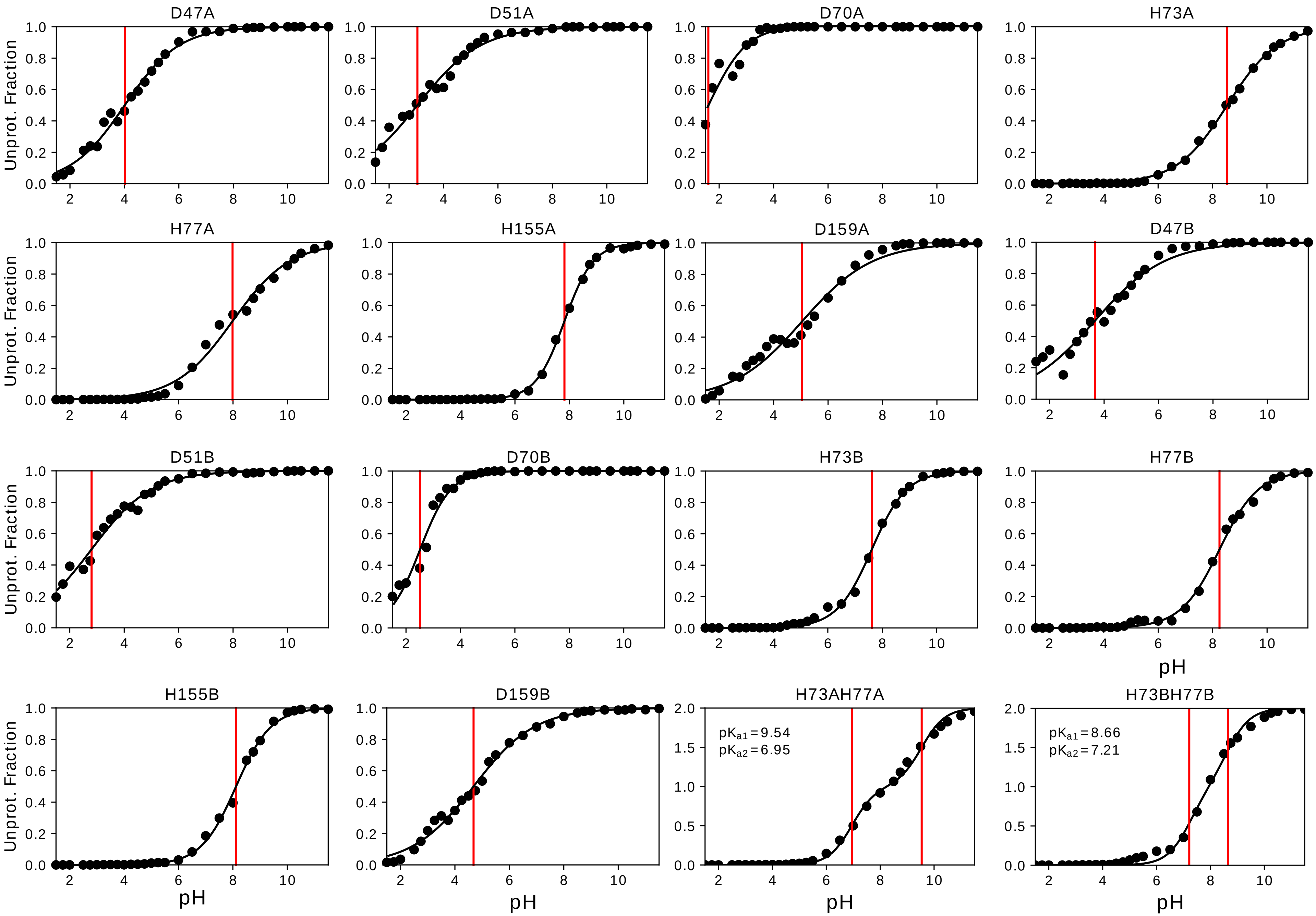

D47A
D51A
D70A
H73A
H77A
H155A
D159A
D47B
D51B
D70B
H73B
H77B
H155B
D159B
H73AH77A
H73BH77B
Unprot. Fraction
pH
$pK_{a1} = 9.54$
$pK_{a2} = 6.95$
$pK_{a1} = 8.66$
$pK_{a2} = 7.21$

Suppl. Fig. 9

bioRxiv preprint doi: https://doi.org/10.1101/2023.02.23.529644; this version posted February 23, 2023. The copyright holder for this preprint (which was not certified by peer review) is the author/funder, who has granted bioRxiv a license to display the preprint in perpetuity. It is made available under aCC-BY 4.0 International license.

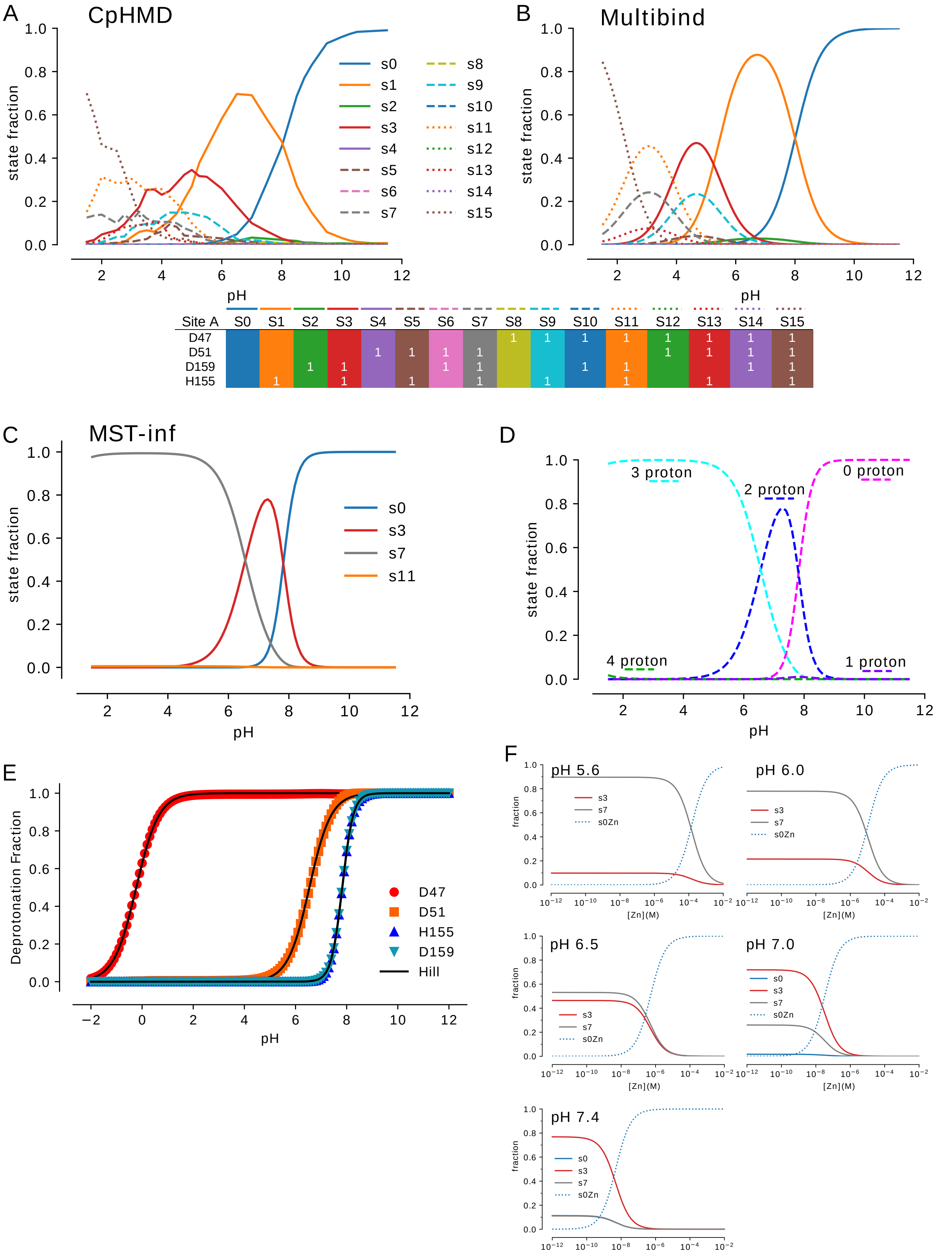

Suppl. Fig. 10

bioRxiv preprint doi: https://doi.org/10.1101/2023.02.23.529644; this version posted February 23, 2023. The copyright holder for this preprint (which was not certified by peer review) is the author/funder, who has granted bioRxiv a license to display the preprint in perpetuity. It is made available under aCC-BY 4.0 International license.

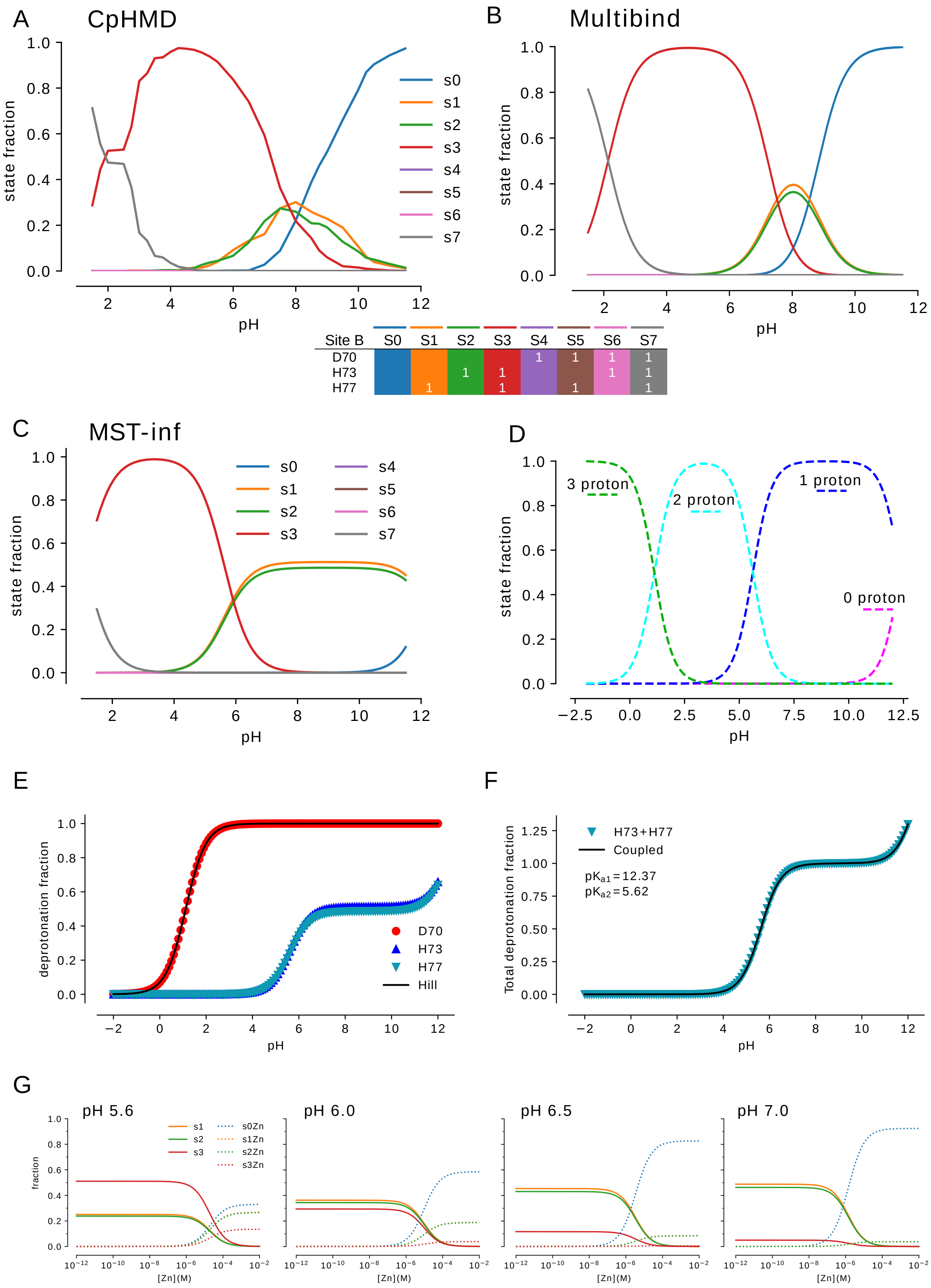

Suppl. Fig. 11

bioRxiv preprint doi: https://doi.org/10.1101/2023.02.23.529644; this version posted February 23, 2023. The copyright holder for this preprint (which was not certified by peer review) is the author/funder, who has granted bioRxiv a license to display the preprint in perpetuity. It is made available under aCC-BY 4.0 International license.

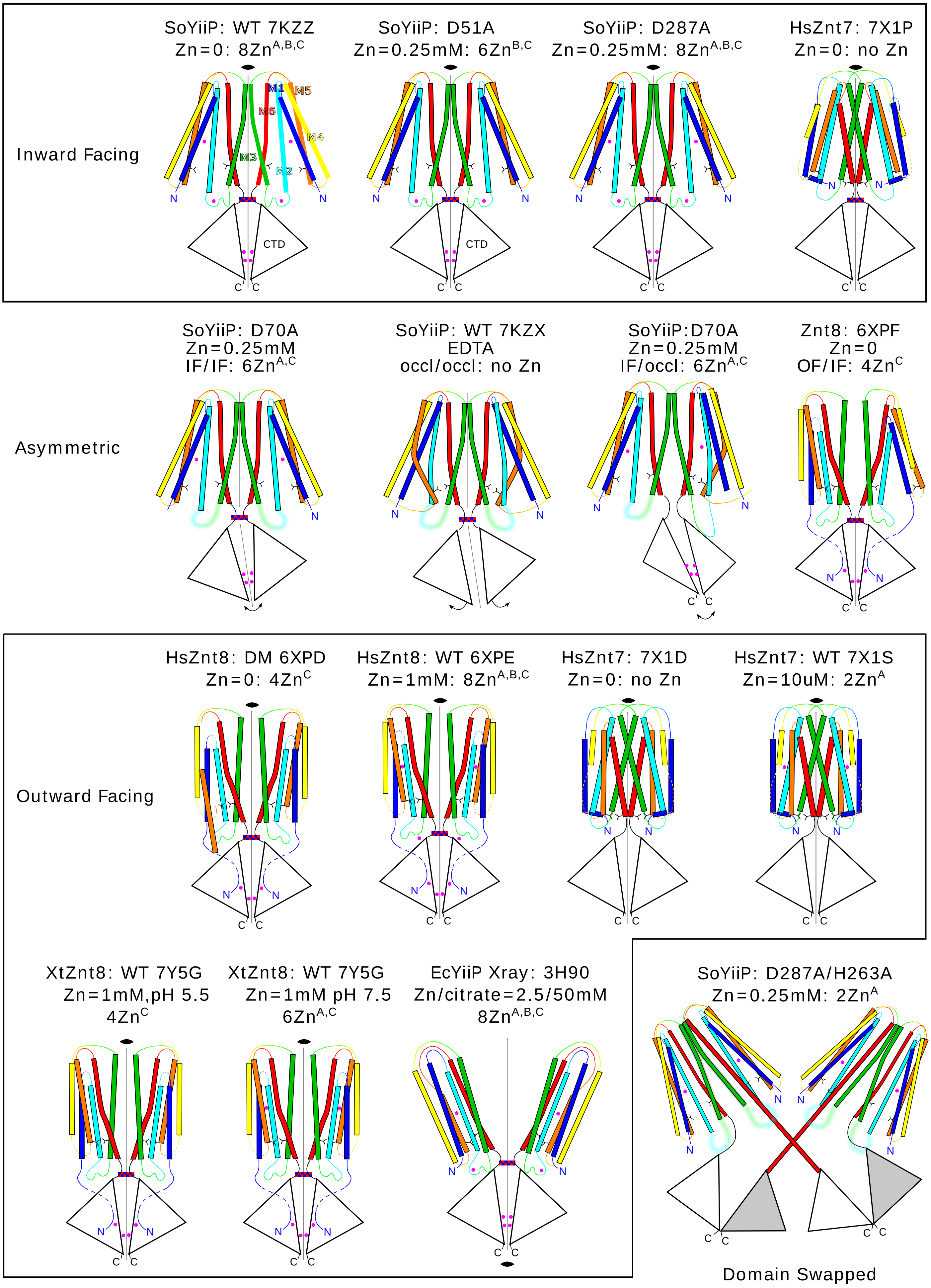

Suppl. Fig. 12

bioRxiv preprint doi: https://doi.org/10.1101/2023.02.23.529644; this version posted February 23, 2023. The copyright holder for this preprint (which was not certified by peer review) is the author/funder, who has granted bioRxiv a license to display the preprint in perpetuity. It is made available under aCC-BY 4.0 International license.

Supplemental Figure Legends

Suppl. Figure 1. Determination of WT and D51A structures by cryo-EM.

(A,B) SDS-PAGE, left, and elution profile from SEC purification. Molecular weight standards are shown on the right at 116, 66, 45, 35, 25, 18.4, 14.4 kDa, with the YiiP monomer running as expected at 32.5 kDa. (C,D) The cryo-EM workflow starts with correction of beam-induced motion and contrast transfer function (CTF) of movies, followed by template-based particle picking. The particle set was subjected to two rounds of 2D classification and ab initio structure determinations to remove false positives. (E,F) Hetero-refinement jobs were used to look for multiple conformations and to maximize the homogeneity of particles for a final non-uniform refinement job, in this case with C2 symmetry. (G,H) The final structure was characterized by local resolution, represented by the colored surface shown from two orthogonal directions, and the Fourier Shell Correlation.

Suppl. Figure 2. Density at Zn^{2+} sites in the cryo-EM maps.

An overview of each cryo-EM structure is shown on the left, followed by densities at sites A, B and C, respectively. Contour levels used to display the various maps were as follows. WT: 7 σ, D51A: 7 σ, D287A, 6.5 σ for sites A and C, 5.5 σ for site B, D70A_asym: 9 σ, D70A_sym: 9 σ, D287A/H263A: 10 σ. Side-chains from selected residues only are shown, for clarity. For D70A_sym, site B is disordered in both protomers. In D70A_asym, the TM2/TM3 loop forms a Zn^{2+}-free association with the CTD (H1 and C-terminus of M6) in one protomer and is disordered in the other protomer. In D287A/H263A, one of the TM2/TM3 loops associates with CTD's from opposing protomers (CTDb, CTDc and TMDd refer to chains B, C and D, respectively). The other TM2/TM3 loop associates with the TM6/CTD linker as shown in Suppl. Fig. 5.

Suppl. Figure 3. Determination of D70A structures by cryo-EM

(A) SDS-PAGE and elution profile for SEC purification; molecular weight markers are at 116, 66, 45, 35, 25, 18.4, 14.4 kDa. The workflow for image processing was complex, involving multiple attempts to segregate distinct classes via ab initio and hetero-refinement jobs. This chart is a summary of the final, productive steps leading to structures presented. (B) Initial steps included correction of beam-induced motion and CTF followed by template-based particle picking, rounds of 2D classification and ab initio structure determination. (C) Hetero-refinement was used to segregate two major classes: class 1 comprising the expected dimer and class 2 comprising a dimer of dimers. The dimer of dimers involved interactions between Fab domains and thus did not appear to affect the conformations adopted by YiiP. (D) Particles from class 1 were subjected to further hetero-refinement to segregate into an additional two classes: one with symmetric TMD's (purple) and the other with asymmetric TMD's (blue) as described in the text. (E) Particles from class 2 (dimer of dimers) were exported to RELION where symmetry expansion and signal subtraction was used to generate a larger set comprising isolated dimers. (F) These symmetry-expanded particles were grouped together with class 1 particles for hetero-refinement using reference volumes with symmetric and asymmetric TMD's as well as a junk collector (not shown). (G, H) The resulting final classes were then used for non-uniform refinement to produce D70A_sym and D70A_asym structures, each colored according to local resolution and shown from orthogonal angles.

bioRxiv preprint doi: https://doi.org/10.1101/2023.02.23.529644; this version posted February 23, 2023. The copyright holder for this preprint (which was not certified by peer review) is the author/funder, who has granted bioRxiv a license to display the preprint in perpetuity. It is made available under aCC-BY 4.0 International license.

Suppl. Figure 4. Determination of D287A and D287A/H263A structures by cryo-EM

(A,B) SDS-PAGE and elution profiles from SEC purifications. Images come from a single gel with the molecular weight markers (116, 66, 45, 35, 25, 18.4, 14.4 kDa). Position and presence of the double peak is consistent with the higher order oligomerization seen during image analysis. (C,D) Corrections for motion and CTF were followed by two rounds of 2D classification and ab initio structure determination. (E,F) Hetero-refinement was used to select a homogeneous set of particles for non-uniform refinement. (G,H) Final structures are characterized by FSC and by local resolution, illustrated by the plots and by surface coloring, respectively.

Suppl. Fig. 5. Interactions between the TM2/TM3 loop and the CTD.

(A) In the WT structure, the TM2/TM3 loop (blue) contacts the TM6/CTD linker from the opposing protomer with proximity of D72 and R210. (B) In the D70A_asym structure, the Zn^{2+}-free TM2/TM3 loop extends to interact with the H1 helix in the CTD of the opposing protomer. (C) In the D287A/H263A structure, the TM2/TM3 loop extends towards CTD's of two different protomers (e.g., TM2/TM3 loop from chain B inserts between CTD's of chains A and D). (D) In the other monomer of D287A/H263A, the TM2/TM3 loop interacts with the TM6/CTD linker of an opposing chain (e.g., the loop from chain A interacts with the linker from chain B). In these chains, the TM6/CTD linker has refolded into one, long continuous helix.

Suppl. Fig. 6. Measuring Zn^{2+} affinity by MST

(A-D) SEC elution profiles for YiiP constructs. WT protein (peak at 12.4 ml) is included for comparison but was not analyzed by MST. The site A mutant, D70A/H263A/D287A, site B mutant, D51A/H263A/D287A, and site C mutant, D51A/D70A, produced peaks at at 12.6 ml, 12.3 ml and 12.6 ml respectively. (E-G) Raw data from MST for the titrations at pH 7 plotted in Fig. 4. The blue rectangles correspond to the time window used for initial fluorescence levels and the red rectangles correspond to the time window used for the thermophoresis rates. Each plot includes all Zn^{2+} concentrations, which were performed in triplicate.

Suppl. Fig. 7. Walk of replica simulations in CpHMD pH-replica exchange (REX) through pH space.

Each panel shows how one replica simulation (Rep:0 to Rep:29) changes over the course of the simulation (Frame number) its current pH state, as indicated by the "pH replica" on the ordinate. The pH replicas range from pH 1.5 (pH replica 0) to 11.5 (pH replica 29). Multiple replicas sample most of the pH range and many move across the especially relevant range between pH replica 9 (pH 4) and pH replica 22 (pH 9), indicating that the REX procedure samples pH space well.

Suppl. Fig. 8. Convergence of the deprotonated fraction for titratable residues in CpHMD simulations.

Residues in site A (D47, D51, H155, D159) and site B (D70, H73, H77) from both protomers (A and B) are shown. The instantaneous deprotonated fraction S is plotted as a function of simulation time and pH value, sampled across all replicas in the replica exchange simulation. The deprotonated fractions generally converge to a stable value after about 8 ns with similar behavior in both protomers A and B, thus indicating sufficient sampling during the CpHMD simulations.

bioRxiv preprint doi: https://doi.org/10.1101/2023.02.23.529644; this version posted February 23, 2023. The copyright holder for this preprint (which was not certified by peer review) is the author/funder, who has granted bioRxiv a license to display the preprint in perpetuity. It is made available under aCC-BY 4.0 International license.

Suppl. Fig. 9. Titration curves for titratable residues in CpHMD simulations.

Residues in site A (D47, D51, H155, D159) and site B (D70, H73, H77) are shown from both protomers (A and B). The unprotonated fraction *S* from the end of the CpHMD simulations is shown as a function of pH (black points). The Hill equation (generalized Henderson-Hasselbalch equation) is fitted to the data (black line). The estimated pK_a is indicated as a red line at S=0.5. As described in the text, H73/H77 were considered to be coupled and were analyzed in aggregate; the two pK_as for the combined system (H73AH77A and H73BH77B) are represented by the pair of red lines in the last two panels.

Suppl. Fig. 10. Site A Protonation and Zn^{2+}-binding states by CpHMD and MST inference.

(A). Populations of protonated states obtained directly from CpHMD simulations for site A, in the absence of Zn^{2+}. Data for protomer A and protomer B were combined. Color coded definitions of individual states are shown below with a "1" indicating the protonated state and absence of a number the unprotonated state of each corresponding residue. (B) Populations of protonated states from an inverse *Multibind* model based on inferring microscopic pK_a values directly from the CpHMD populations in (A). (C) Population of protonated states resulting from MST inference, which relies on the MC method to refine microscopic model parameters based on experimental MST data. Populations represent the Zn^{2+}-free states although the model incorporates the whole range of Zn^{2+} bound states [see (F)]. Only states S0, S3, S7, S11 have appreciable (>0.0001 maximum probability) although all states contribute to aggregated state probabilities in D and E. (D) MST inference results represented as populations of aggregated states defined by the total number of protons bound in the absence of Zn^{2+}. (E) Deprotonated fraction of each site A residue as a function of pH, averaged over all possible states of the MST inference model in the absence of Zn^{2+}. Data points (symbols) were generated from the model; then the Hill-Langmuir equation was fit to the generated data in order to obtain an effective per-residue pK_a (listed in Table 4). (F) Population of states derived from the MST inference model as a function of Zn^{2+} concentration at various pH values. S0Zn (dotted line) denotes the fully deprotonated, Zn^{2+}-bound state, which is the only populated Zn^{2+}-bound state predicted by the MST inference.

Suppl. Fig. 11. Site B Protonation and Zn^{2+}-binding states by CpHMD and MST inference.

(A). Populations of protonated states obtained directly from CpHMD simulations for site B, in the absence of Zn^{2+}. Data for protomer A and protomer B were combined. Color coded definitions of individual states are shown below with a "1" indicating the protonated state and absence of a number the unprotonated state of each corresponding residue. (B) Populations of protonated states from an inverse *Multibind* model based on inferring microscopic pK_a values directly from the CpHMD populations in (A). (C) Population of protonated states resulting from MST inference. For this analysis, a symmetry restraint was imposed on H73 and H77 as described in the text. Populations are shown in the absence of Zn^{2+} although the model incorporates the whole range of Zn^{2+} concentrations [see (G)]. (D) MST inference results represented as populations of aggregated states defined by the total number of protons bound in the absence of Zn^{2+}. (E) Deprotonated fraction of each site B residue as a function of pH, averaged over all possible states of the MST inference model in the absence of Zn^{2+}. Data points were generated from the model and the Hill-Langmuir equation was fit to D70 data (solid black line) to obtain an effective per-residue pK_a; data for H73 and H77 are not correctly modelled by a Hill fit (not plotted). (F) H73 and H77 were treated as a coupled system and the total deprotonated fraction as a function of pH was fit with the “coupled titration model” (solid black line), resulting in two

bioRxiv preprint doi: https://doi.org/10.1101/2023.02.23.529644; this version posted February 23, 2023. The copyright holder for this preprint (which was not certified by peer review) is the author/funder, who has granted bioRxiv a license to display the preprint in perpetuity. It is made available under aCC-BY 4.0 International license.

effective pK_a values. (G) Population of states from the MST inference mode as a function of Zn^{2+} concentration at various pH values. Unlike site A, multiple Zn^{2+}-bound states (S0Zn, S1Zn, S2Zn, S3Zn) are populated at lower pH values.

Suppl. Fig. 12. Summary of structures from CDF transporters.

Cartoon representations of most structures so far determined. The structures have been grouped according to conformational state: symmetric IF, symmetric OF and asymmetric states. PDB accession codes are indicated for previously published structures together with concentrations of Zn^{2+} present in the solution and the number of ions observed in the structures. Accession codes for current work are listed in Table 1. Although the sites been named differently for Znt7 and Znt8, the A,B,C nomenclature has been used to simplify the comparison. The helices in the TMD are shown in rainbow colors starting with blue for TM1 and red for TM6. CTD's are represented as a triangle. Zn^{2+} ions as magenta spheres. Hydrophobic residues forming the so-called hydrophobic gate (Leu154 and Leu199 in SoYiiP) are shown as sticks. Disordered in TM2/TM3 loops is indicated by a blurred line.
